# Toward a Medical Gastric Bypass: Chronic Feeding Studies With Liraglutide + PYY_3-36_ Combination Therapy in Diet-Induced Obese Rats

**DOI:** 10.3389/fendo.2020.598843

**Published:** 2021-01-22

**Authors:** Ulrich Dischinger, Julia Hasinger, Malina Königsrainer, Carolin Corteville, Christoph Otto, Martin Fassnacht, Mohamed Hankir, Florian Johannes David Seyfried

**Affiliations:** ^1^Department of Internal Medicine, Division of Endocrinology and Diabetes, University Hospital, University of Würzburg, Würzburg, Germany; ^2^Department of General, Visceral, Transplant, Vascular and Pediatric Surgery, University Hospital, University of Würzburg, Würzburg, Germany

**Keywords:** obesity, rygb, liraglutide, peptide tyrosine tyrosine (PYY), treatment, gastric bypass, peptide tyrosine tyrosine 3-36 (PYY_3-36_)

## Abstract

**Background:**

Combination therapies of anorectic gut hormones partially mimic the beneficial effects of bariatric surgery. Thus far, the effects of a combined chronic systemic administration of Glucagon-like peptide-1 (GLP-1) and peptide tyrosine tyrosine 3-36 (PYY_3-36_) have not been directly compared to Roux-en-Y gastric bypass (RYGB) in a standardized experimental setting.

**Methods:**

High-fat diet (HFD)-induced obese male Wistar rats were randomized into six treatment groups: (1) RYGB, (2) sham-operation (shams), (3) liraglutide, (4) PYY_3-36_, (5) PYY_3-36_+liraglutide (6), saline. Animals were kept on a free choice high- and low-fat diet. Food intake, preference, and body weight were measured daily for 4 weeks. Open field (OP) and elevated plus maze (EPM) tests were performed.

**Results:**

RYGB reduced food intake and achieved sustained weight loss. Combined PYY_3-36_+liraglutide treatment led to similar and plateaued weight loss compared to RYGB. Combined PYY_3-36_+liraglutide treatment was superior to PYY_3-36_ (p ≤ 0.0001) and liraglutide (p ≤ 0.05 or p ≤ 0.01) mono-therapy. PYY_3-36_+liraglutide treatment and RYGB also reduced overall food intake and (less pronounced) high-fat preference compared to controls. The animals showed no signs of abnormal behavior in OF or EPM.

**Conclusions:**

Liraglutide and PYY_3-36_ combination therapy vastly mimics reduced food intake, food choice and weight reducing benefits of RYGB.

## Introduction

Obesity nowadays affects more than one-third of human individuals and represents a major escalating socioeconomic burden (1). Moreover, obesity correlates with elevated risk for several serious, life-shortening diseases, such as diabetes, chronic heart failure, cardiovascular disease, and several types of cancer (2).

Available non-invasive treatment options for severe obesity lack efficacy and result in long-term weight regain in the vast majority of cases. In contrast, bariatric surgery, in particular Roux-en-Y gastric bypass (RYGB), achieves marked and sustained weight loss but also improves metabolic health (3–6). The persistently negative energy balance after surgery is considered to be largely due to a decrease in energy intake (approximately 50%) (7–20), mostly from fat (7, 10, 12, 15, 17, 18, 20–22).

Circulating levels of the appetite-suppressing anorectic and glucoregulatory gut hormones derived from enteroendocrine L-cells such as glucagon-like peptide-1 (GLP-1) and peptide tyrosine tyrosine 3-36 (PYY_3-36_) are markedly increased after Roux-en-Y gastric bypass surgery (RYGB) (23–32). However, studies in which signaling of the GLP-1 receptor or the PYY_3-36_-preferring Y2 receptor are individually perturbed by genetic or pharmacologic means in RYGB-operated humans or rodents have generally shown little to no influence on food intake or body weight (33–37). It was consequently proposed that GLP-1 and PYY_3-36_ may compensate for each other when either one is blocked after RYGB, but this has not received support in mice with combined deletion of the GLP-1 receptor and the Y2 receptor (38). Thus, significant doubt has been cast on the causal roles of GLP-1 and PYY_3-36_ in the long-term negative energy balance induced by RYGB.

This does, however, not necessarily undermine the potential of GLP-1 and PYY_3-36_ as anti-obesity treatments. It has been shown that GLP-1 and PYY_3-36_ reduce food intake additively in rodents and humans when administered exogenously in an acute setting (39), while chronic administration of the clinically approved GLP-1 analogue liraglutide with PYY_3-36_ additively reduce body weight in diet-induced obese hamsters—similar to the patterns found after RYGB (40). We previously found that the anorectic gut hormones GLP-1 and PYY_3-36_ reduce high-fat food preference additively after RYGB in diet-induced obese rats (41).

These findings still suggest that GLP-1 + PYY_3-36_ combination therapy holds genuine promise as a noninvasive alternative to RYGB for treating obesity, although these peptides are probably not the only or main factors for the effects and actions of RYGB. However, the short half-life of PYY_3-36_ of only 4 h prevents it clinical use (42). GLP-1 is even more rapidly degraded to inactive metabolites, resulting in a half-life of only 10 min (43). Synthetic, long-acting GLP-1 receptor agonists like liraglutide (half-life in humans approximately 12 h), however, are used for the successful treatment of obesity (44, 45).

Thus, to formally test the combined effects of liraglutide and PYY_3-36_ in a standardized obesity model and, thereby, show the potential of a combinatory medical treatment for obesity, we directly compared the effects of RYGB with daily subcutaneous liraglutide injections in combination with PYY_3-36_ delivered by subcutaneous osmotic mini-pump on food intake, food choice, and body weight in diet-induced obese Wistar rats. To control for abnormal behavioral changes under these substances, which might especially occur during PYY_3-36_ treatment (46), open field and elevated plus maze test were performed. To assess the effects on body composition, we analyzed the weights of retroperitoneal and epididymal white adipose tissue (WAT) depots *ex vivo*.

## Methods

### Animals

As published before (41), adult male Wistar rats (Charles River Laboratories, *n* = 58) weighing 329.8 ± 2.2 g, 9–10 weeks old were initially group-housed in a dedicated facility with an ambient room temperature of 22°C and a 12 h light/dark cycle. They had free access to a High Fat Diet (C1090-60 HF diet, 5,228 kcal/kg; 60% calories from fat, 16% from protein, and 24% from carbohydrate; Altromin) for about 6 weeks to induce obesity. The animals were randomized into the following pharmaceutical/surgical treatment groups: Five animals received liraglutide s.c. (0.4 mg/kg/day, Victoza, Novo Nordisk Pharma) and isotonic saline (Braun) *via* osmotic minipump. Five animals received PYY_3-36_ (0.1 mg/kg/day, Hölzel diagnostika) *via* osmotic minipump and saline s.c. Eleven animals received a combination of liraglutide s.c. and PYY_3-36_ *via* osmotic minipump and nine animals received saline only (*via* osmotic minipump and s.c.). Fifteen animals underwent RYGB and thirteen animals a sham operation. Generally, we attended the principle of the 3Rs, that the number of animals necessary is the minimum required for statistical significance based on effect sizes and results. Based on our previous experiences (47), a number of 5–7 animals is necessary for this. Usually, we calculate for a dropout rate of 30% in our RYGB model and 5% in our sham model. For some reason, we experienced a far higher dropout rate (50%) during a previous study and thus we decided to strengthen these particular groups.

All animal procedures were approved by the local regulatory authority (Regierung von Unterfranken, Würzburg, Germany, AZ: 55.2-2532-2-467). All experiments were performed in accordance with German and European laws and regulations (TierSchG, TierSchVersV, Directive 2010/63/EU).

### Drugs

Liraglutide was administered subcutaneously at a dose of 0.4 mg/kg/day once daily. PYY_3-36_ was given at a dose of 0.1 mg/kg/day (40, 48). To overcome the short half-life of PYY_3-36_ (42), it was administered *via* osmotic minipump (ALZET pump model 2004). Osmotic minipumps were implanted subcutaneously into the interscapular region of the animals under isoflurane anesthesia with butorphanol analgesia and postoperative carprofen analgesia (one injection of 5 mg/kg carprofen). Isotonic saline was administered at a dose of 1 µl/g body weight once daily subcutaneously (corresponding to the volume of liraglutide) and/or *via* osmotic minipump.

### Surgeries

For RYGB and sham operation, the animals were isoflurane-anesthetized and under butorphanol (0.1 mg/kg) analgesia. Briefly, for the RYGB operation a small gastric pouch 5% of the original stomach size was created and the biliopancreatic and common limbs were made to measure 15 and 25 cm in length, respectively (41, 47). Animals were then transferred to individual cages and for the first 2 postoperative days were only given a liquid diet (chocolate-flavored Ensure, a nutrition drink, 0.93 kcal/ml; 22% calories from fat) and carprofen analgesia. Postoperatively, all animals received carprofen (5 mg/kg) for 5 days. From postoperative day 3 onwards, animals were maintained on a choice of C1090-10 LF (3,514 kcal/kg; 10% calories from fat, 24% from protein, and 66% from carbohydrate; Altromin) and HF diets. LF and HF diet intakes were measured daily until day 24 for each animal and HF/LF food preference was calculated by dividing the daily calories consumed from the HF/LF diet by the total daily calories consumed from both diets and expressed as percentage.

### Open Field

The open field (OF) is used to test for locomotor activity, exploratory behavior, and anxiety (49). Normally, rodents avoid open areas and present thigmotaxis, the degree depending on their anxiety level. As OF, an apparatus of polyethylene with a square base (1 m²) and 60 cm high sidewalls was used. The animals were transferred to the center of this box individually and their activity was recorded for 10 min immediately after this. After each run, the OF was cleaned with Terralin liquid. At a later time point, the recordings were analyzed. The time in the central area (central square, 90 × 90 cm) of the OF, the number of entries in this area, as well as the rearing behavior was evaluated. Animals were tested at about 2 weeks after intervention.

### Elevated Plus Maze

The elevated plus maze (EPM) is used for the evaluation of anxiety (50). As EPM, an elevated (1 m) apparatus of polyethylene with a central area (10 cm²) with four attached arms (50 × 10 cm, two opposing arms with 30 cm sidewalls) was used. The animals were transferred to the center of the EPM individually and their activity was recorded for 5 min immediately after this. After each run, the EPM was cleaned with Terralin liquid. At a later time point, the recordings were analyzed. The time in the open arms of the EPM and the number of entries in the open arms was measured. Animals were tested at about 2 weeks after intervention.

### *Ex Vivo* White Adipose Tissue Analysis

After a midline laparotomy was performed and the intestine removed, the retroperitoneum was exposed. As described before, we chose the aorta as medial, the fascia transversalis as lateral and the psoas muscle as dorsal dissection border (51). Directly following dissection, retroperitoneal and epididymal (rWAT and eWAT) fat pads were weighed using a precision balance.

### Statistical Analysis

Two-tailed unpaired t-test and two-way ANOVA with Tukey’s *post-hoc* comparison test where appropriate were used for statistical analysis using GraphPad Prism (Version 8.1.2) software. Due to inexplicably high weight loss, one RYGB treated animal had to be excluded from all analyses.

## Results

### Roux-En-Y Gastric Bypass and Peptide Tyrosine Tyrosine 3-36 +Liraglutide Lead to Similar Sustained Plateaued Body Weight

**Figure 1:**
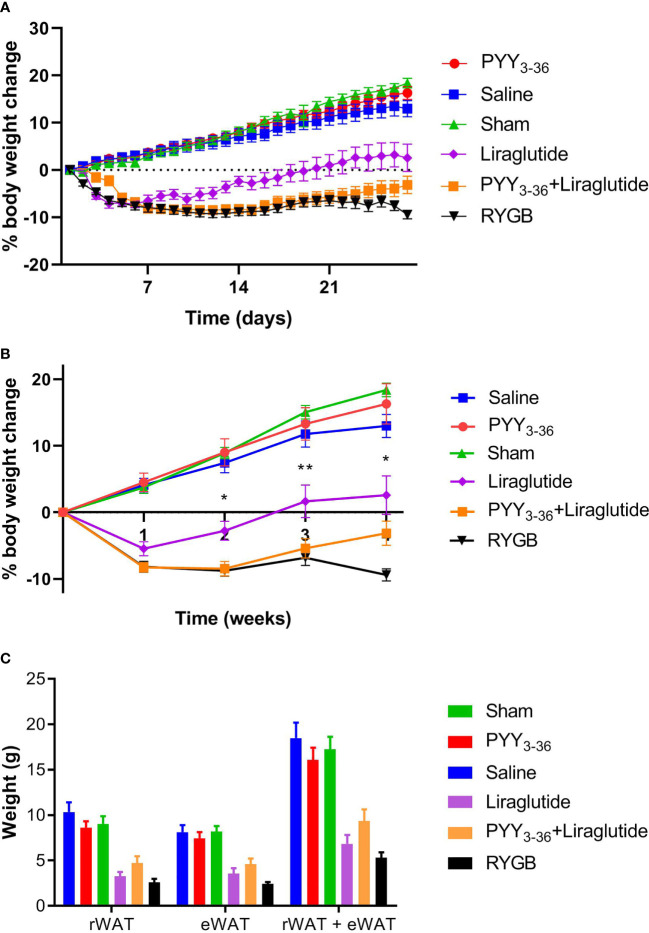
RYGB and PYY_3-36_+liraglutide lower body weight in diet-induced obese Wistar rats. Body weight in percent (%) weight change from baseline (intervention) of RYGB (n = 14), sham (n = 13), PYY_3-36_+liraglutide (n = 11), liraglutide (n = 5) and saline (n = 9) groups. **(A)** daily and **(B)** weekly. *p ≤ 0.05 and **p ≤ 0.01 for differences between PYY_3-36_+liraglutide and liraglutide as determined by two-way ANOVA with Tukey’s *post-hoc*. **(C)** Weights of rWAT, eWAT and sum of rWAT+eWAT. Significant differences between PYY_3-36_+liraglutide, liraglutide, RYGB, and their respective control group (not shown). **(A–C)** Data are presented as mean ± standard error of the mean.

On the day of surgery and start of medical treatment, respectively, animals of the different treatment/control groups had similar body weights (477.7 ± 8.4 g for the RYGB group, 479.2 ± 11 g for the sham group, 547.8 ± 19.5 g for the PYY_3-36_ +liraglutide group, 502.4 ± 7.6 g for the PYY_3-36_ group, 487.6 ± 9.6 g for the liraglutide group, and 527.1 ± 18.6 g for the saline group). The weight course after intervention is shown in **Figure 1**. For better visualization of the differences, statistics were performed for weekly intervals. Post intervention, shams, saline, and PYY_3-36_ treated animals gained weight continuously, while RYGB, PYY_3-36_ +liraglutide and liraglutide treated animals started to lose weight. RYGB and PYY_3-36_ +liraglutide treated animals achieved sustained and plateaued weight loss, while the other two groups started to regain weight upon the second week during the further observation period of 4 weeks (effect of intervention: *F*_(5, 255)_ = 221.6, *p* ≤ 0.0001; effect of time: *F* _(4, 255)_ = 43.48, p ≤ 0.0001; interaction: *F*_(20, 255)_ = 18.11, *p* ≤ 0.0001. **Figure 1B**).

The *post-hoc* test reveals a significant difference between RYGB and sham from the first week post intervention onwards (p ≤ 0.0001). A significant difference between PYY_3-36_+liraglutide and saline (p ≤ 0.0001), as well as between liraglutide and saline (p ≤ 0.0001) treated animals was also seen from the first week post intervention. A significant difference between PYY_3-36_ +liraglutide and liraglutide only was detected from the second week onwards (p ≤ 0.05 or p ≤ 0.01). Liraglutide treated animals also differed from RYGB treated from week two onwards (p ≤ 0.05 and p ≤ 0.0001). There was no significant difference between PYY_3-36_ and saline at any time point.

### Roux-En-Y Gastric Bypass and Peptide Tyrosine Tyrosine 3-36 +Liraglutide Lower Overall Food Intake and Preference for High-Fat Diet

**Figure 2:**
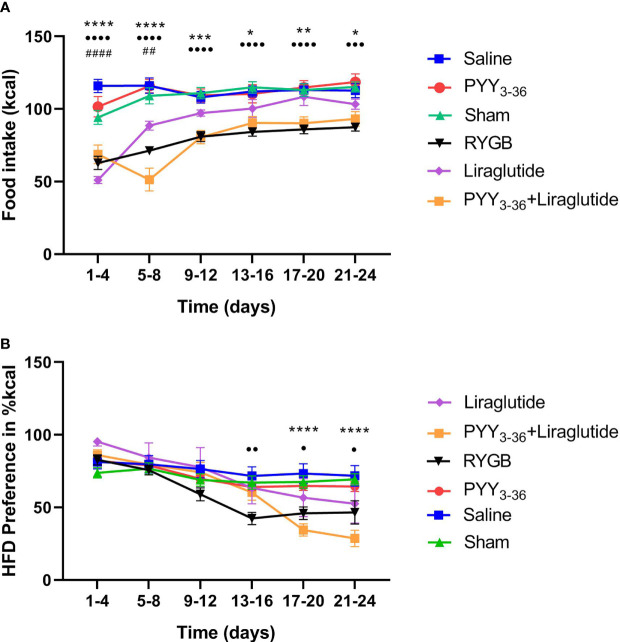
RYGB and PYY_3-36_+liraglutide lower overall food intake. **(A)** Food intake in kcal and **(B)** preference for high-fat diet in percent (kcal) in 4-day intervals of RYGB (n = 14), sham (n = 13), PYY_3-36_+liraglutide (n = 11), liraglutide (n = 5), PYY_3-36_ (n = 5), and saline (n = 9) groups post intervention. **(A)** ••••p ≤ 0.0001 and •••p ≤ 0.001 for differences between RYGB and Sham; ****p ≤ 0.0001, ***p ≤ 0.001, **p ≤ 0.01, *p ≤ 0.05 for differences between PYY_3-36_+liraglutide and saline; ^####^p ≤ 0.0001, ^##^p ≤ 0.01 for differences between liraglutide and saline as determined by two-way ANOVA with Tukey’s *post-hoc*. **(B)** •p ≤ 0.05 and ••p ≤ 0.01 for differences between RYGB and Sham and ****p ≤ 0.0001 for difference between PYY_3-36_+liraglutide and saline as determined by two-way ANOVA with Tukey’s *post-hoc*. **(A, B)** Data are presented as mean ± standard error of the mean.

In order to study the effects of RYGB, PYY_3-36_ and liraglutide and a combination of both on food intake and food choice, animals from both surgical groups were given free access to LF (10% calories from fat) and HF (60% calories from fat) diets from the third postoperative day onwards. RYGB, PYY_3-36_+liraglutide, and liraglutide led to a reduced overall food intake (in kcal) in the observation period (effect of intervention: *F*_(5, 300)_ = 75.2, *p* ≤ 0.0001; effect of time: *F*_(5, 300)_ = 17.3, p ≤ 0.0001; interaction: *F*_(25, 300)_ = 3.3, *p* ≤ 0.0001. **Figure 2A**). *Post-hoc* testing shows, that animals of the PYY_3-36_+liraglutide group consumed significantly less food than saline (p ≤ 0.05, p ≤ 0.01, p ≤ 0.001, and p ≤ 0.0001) and RYGB treated animals consumed significantly less than shams (p ≤ 0.001 and p ≤ 0.0001) throughout the whole observation period, while liraglutide treated animals only differed from saline treated in intervals 1 and 2. Moreover, there was a significant difference between PYY_3-36_+liraglutide and liraglutide regarding food intake at interval 2. Regarding food preference, the effects of the intervention were less pronounced (**Figure 2B**). RYGB treated animals showed a significantly reduced preference for 60% fat diet compared to sham treated animals in intervals 4, 5, and 6 (p ≤ 0.01 and p ≤ 0.05), PYY_3-36_ +liraglutide treated animals differed from saline treated in interval 5 and 6 (p ≤ 0.0001).

### No Abnormal Behavior Was Found Under Any Treatment

**Figure 3:**
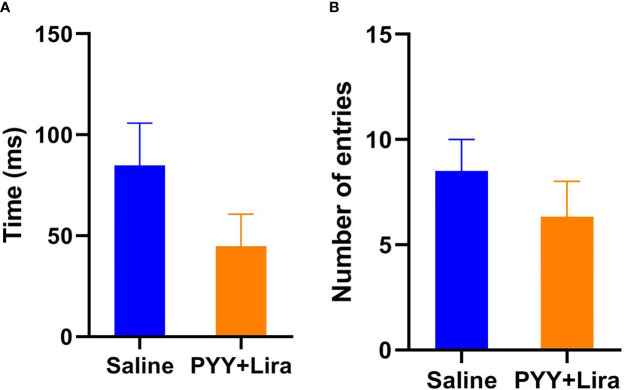
Open field, time in central area. **(A)** Time (in ms) of the animals in the central area of the open field. **(B)** Number of entries into the central area of the open field. PYY_3-36_+liraglutide n = 6, saline n = 4. Data are presented as mean ± standard error of the mean.

During open-field tests no significant differences between RYGB, sham, saline, and PYY_3-36_ +liraglutide treated animals were found in the open field regarding time in the central area (*F*_(5, 35)_ = 0.65, p = 0.66; **Figure 3**) or entries into the central area (*F*_(5, 35)_ = 1.28, p = 0.29).

No significant differences between RYGB, sham, saline, and PYY_3-36_ +liraglutide treated animals were found in the elevated plus maze regarding time in the open arms (*F*_(5, 35)_ = 3.62, p = 0.0096; no sig. difference between RYGB and sham or saline and PYY_3-36_ +liraglutide in the *post-hoc* test) or entries into the open arms (*F*_(5, 35)_ = 1.02, p = 0.42).

### Roux-En-Y Gastric Bypass and Peptide Tyrosine Tyrosine 3-36+Liraglutide Leads to Similar Reductions of Retroperitoneal and Epididymal Fat Pads

Weights of rWAT and eWAT were significantly lower in RYGB, PYY_3-36_ +liraglutide and liraglutide treated animals compared to their respective control group (*F*_(5, 50)_ = 22.4, *p* ≤ 0.0001). Comparing amounts of eWAT+rWAT, no differences were found between RYGB, PYY_3-36_ +liraglutide and liraglutide treated animals (**Figure 1C**).

## Discussion

The mechanisms behind the weight lowering effect of RYGB are not entirely understood. It has been repeatedly shown, however, that meal-stimulated levels of the enteroendocrine hormones peptide tyrosine tyrosine 3-36 (PYY_3-36_), glucagon-like peptide-1 (GLP-1), and other L-cell derived hormones are remarkably increased after RYGB (23–32). The relevance of their changed secretion for the effectivity of RYGB seems convincing but is still unclear, as well as their synergistic effectivity for the treatment of obesity, which we aimed to examine. Studies in which signaling of the GLP-1 receptor or the PYY_3-36_-preferring Y2 receptor are perturbed after RYGB have generally shown little to no influence on food intake or body weight (33–37). Additionally, RYGB increases plasma levels of various other anorexigenic gut hormones including oxyntomodulin (52, 53) and neurotensin (54–56), which also reduce food intake. In this sense, a combination therapy with GLP-1, oxyntomodulin and PYY has been shown to achieve superior glucose tolerance and reduced glucose variability compared with RYGB, while its effect on body weight was also less pronounced (52).

In the present study, we directly compared the effects of RYGB and liraglutide + PYY_3-36_ combination therapy on food intake, food choice, and body weight in diet-induced obese rats. We found further evidence that a combinatory treatment with PYY_3-36_ and a GLP-1 agonist (liraglutide) can exert an additive effect on body weight loss similar to that of RYGB, which further underlines their possible usability and efficacy for obesity treatment. The dose of PYY_3-36_ selected in the present study was the same as that used in a previous study that suppressed food intake in diet-induced obese rats (57). However, a 10-fold higher dose of PYY_3-36_ (in combination with liraglutide) was used before in hamsters (40). It would therefore be worth testing higher doses of PYY_3-36_ in combination with liraglutide in future head-to-head comparisons although any potentially adverse side effects would have to be carefully monitored.

As expected, RYGB treated animals lost weight after intervention reaching a stable plateau, while sham treated animals gained body weight continuously (**Figures 1A, B**), leading to highly significant differences over all weeks post intervention. PYY_3-36_+liraglutide treated animals showed a very similar food intake and body weight course. Showing the additive effect of the combinatory treatment, PYY_3-36_+liraglutide treated animals were significantly lighter than liraglutide-only treated animals from week 2 on. Instead, animals, who received PYY_3-36_ only, showed no significant differences compared to saline treated animals regarding food intake, preference, behavior, or metabolic parameters. Although, the mentioned superiority of PYY_3-36_+liraglutide over liraglutide strongly indicates that the route of the PYY_3-36_ application and the substance itself have worked. The detected additive effect might be caused by the manipulation of two different hormonal routes, which are known to reduce food intake independently. If only one route is addressed pharmaceutically, there might be an upregulation of the other and vice versa. Although, this hypothesis would need further confirmation.

The body weight course reflects in measured eWAT and rWAT weights. In this sense, the amount of eWAT+rWAT is significantly lower in PYY_3-36_+liraglutide, liraglutide, RYGB treated animals compared to their respective control group (**Figure 1C**). It has been shown, that measurements of fat pads *ex-vivo* correlate well with *in-vivo* methods like dual-energy X-ray absorptiometry (58). It has been repeatedly shown that RYGB leads to a reduced energy intake (7–20), as it changes food preference decreasing the intake of calories from fat (7, 10, 12, 15, 17, 18, 20–22). In accordance with this, we have recently found that GLP-1 and PYY_3-36_ reduce high-fat food preference additively after RYGB in diet-induced obese rats and that these animals had an increased meal-induced GLP-1 and PYY_3-36_ release (41). In this study, animals of the RYGB and PYY_3-36_+liraglutide group consumed significantly less food than sham treated animals over the whole observation period after intervention (**Figure 2A**). Moreover, RYGB treated animals showed a significantly reduced preference for 60% fat diet compared to sham treated animals (**Figure 2B**) which was also the case for PYY_3-36_+liraglutide treated animals compared to saline in the end of the observation period.

It would be interesting to assess if overlapping or different neural pathways are recruited by RYGB and PYY_3-36_+liraglutide combination therapy to reduce preference for high-fat food, which could include hypothalamic and striatal signaling in future studies. This would be even more interesting, as the onset of changes in HFD preference do not overlap in RYGB and PYY_3-36_+liraglutide treated animals, which might indicate different or additional neural mechanisms.

No significant differences were found in the behavioral tests performed, although, PYY_3-36_+liraglutide treated animals showed some reduced activity in the open field (**Figure 3**). This could be due to a known anxiogenic effect of NPY Y2 receptors, at which PYY_3-36_ mainly mediates its central effects (46). However, the more sensitive EPM did not confirm this observation, which might be therefore less relevant. Altogether, the behavioral tests show no relevant abnormal behavior during medical treatment. This is an important observation, as the fact, that a declining preference for HFD as well as a drop in overall food intake in the second interval (not accompanied by a reduced HFD preference) was shown in PYY_3-36_+liraglutide treated animals, could imply a certain sickness. All in all, however, we found no hints (especially not for visceral malaise), that this might have been the case over the whole observation period. In contrary, PYY_3-36_+liraglutide treated animals plateaued body weight and slightly regained weight in the end of the observation period, which contradicts general sickness as well as serious nausea or vomiting (which were not detectable clinically). Additionally, it is well described that malaise usually occurs early on during the treatment with liraglutide, possibly due to a strong activation of neurons in the area postrema. This in itself is not a learning process and is simply an acute pharmacological effect, which would be expected to desensitize with repeated treatments rather than worsen. To show the safety and tolerability of the treatment, additional behavioral tests at later stages of the treatment period should be performed in further studies. As a limitation, the groups liraglutide and PYY_3-36_ only consisted of only five animals each. Although it has to be mentioned, that the in-group consistency of the mentioned effects was strong. Moreover, our previous experience with RYGB studies clearly suggests that a number of five animals per group is generally sufficient (59–61). However, better-powered and equal group sizes in future studies will allow us to more definitively compare each pharmacological regimen to each other and to RYGB in a standardized setting. Due to unforeseen circumstances, it was not possible to start the individual treatment of all animals on the same day after randomization. This led to the limitation, that RYGB treated animals were significantly lighter than the animals of the PYY_3-36_+liraglutide group. Although this surely weakens the comparability of the groups to some extent, we still feel that the similar body weight change in percent shows the comparable effectivity of the treatments.

In summary, we demonstrated for the first time in a direct head-to-head comparison, that a combination of PYY_3-36_ and liraglutide had similar effects on body weight and food intake compared to RYGB. This strengthens their possible role as parts of a combinatory therapy for obesity. Chronic interventions with a combination of GLP-1 agonists and PYY_3-36_ seem to be very promising. From our observations we surely are not able to conclude, that GLP-1 and PYY_3-36_ are the main factors for the efficacy of RYGB.

## Data Availability Statement

The datasets presented in this study can be found in online repositories. The names of the repository/repositories and accession number(s) can be found below: https://github.com/Dischinger/Towards-a-medical-gastric-bypass.git.

## Ethics Statement

The animal study was reviewed and approved by Regierung von Unterfranken, Würzburg, Germany.

## Author Contributions

UD, MH, FS, and MF planned the experiments. UD, MH, and FS conducted the experiments. FS and CC performed the surgeries. JH, MK, and CO supported the *in vivo* experiments. UD, MH, and FS wrote the manuscript. All authors discussed the results and commented on the manuscript. All authors contributed to the article and approved the submitted version.

## Funding

The research in this manuscript was funded by the IZKF (grant number IZKF Z-2/71).

## Conflict of Interest

The authors declare that the research was conducted in the absence of any commercial or financial relationships that could be construed as a potential conflict of interest.
